# Macrophage Activities in Myocardial Infarction and Heart Failure

**DOI:** 10.1155/2020/4375127

**Published:** 2020-04-22

**Authors:** Sophia Esi Duncan, Shan Gao, Michael Sarhene, Joel Wake Coffie, Deng Linhua, Xingru Bao, Zhang Jing, Sheng Li, Rui Guo, Jing Su, Guanwei Fan

**Affiliations:** ^1^First Teaching Hospital of Tianjin University of Traditional Chinese Medicine, Tianjin, China; ^2^State Key Laboratory of Modern Chinese Medicine, Tianjin University of Traditional Chinese Medicine, Tianjin, China; ^3^Tianjin Laboratory of Translational Research of TCM Prescription and Syndrome, Tianjin 300193, China

## Abstract

Heart diseases remain the major cause of death worldwide. Advances in pharmacological and biomedical management have resulted in an increasing proportion of patients surviving acute heart failure (HF). However, many survivors of HF in the early stages end up increasing the disease to chronic HF (CHF). HF is an established frequent complication of myocardial infarction (MI), and numerous influences including persistent myocardial ischemia, shocked myocardium, ventricular remodeling, infarct size, and mechanical impairments, as well as hibernating myocardium trigger the development of left ventricular systolic dysfunction following MI. Macrophage population is active in inflammatory process, yet the clear understanding of the causative roles for these macrophage cells in HF development and progression is actually incomplete. Long ago, it was thought that macrophages are of importance in the heart after MI. Also, though inflammation is as a result of adverse HF in patients, but despite the fact that broad immunosuppression therapeutic target has been used in various clinical trials, no positive results have showed up, but rather, the focus on proinflammatory cytokines has proved more benefits in patients with HF. Therefore, in this review, we discuss the recent findings and new development about macrophage activations in HF, its role in the healthy heart, and some therapeutic targets for myocardial repair. We have a strong believe that there is a need to give maximum attention to cardiac resident macrophages due to the fact that they perform various tasks in wound healing, self-renewal of the heart, and tissue remodeling. Currently, it has been discovered that the study of macrophages goes far beyond its phagocytotic roles. If researchers in future confirm that macrophages play a vital role in the heart, they can be therapeutically targeted for cardiac healing.

## 1. Introduction

Despite various significant pharmacological progress, heart failure (HF) still has a high morbidity and mortality rate. It occurs when the heart is unable to pump adequate blood and oxygen supply to various parts of the body. Myocardial infarction (MI) can lead to heart failure in several ways; thus, an inadequate supply of oxygen to the heart causes the heart muscle inability to contract well leading to a decrease in the stroke volume (amount of blood pumped from the left ventricle per beat) which may result in congestive heart failure. Generally, HF increases in the aging population [1]. In the USA, approximately 6.5 million adults are suffering from HF, and based on these data, projected 8 million adults are bound to be living with this syndrome by 2030 [2, 3]. Existing data on HF in recent times are approximately 26 million adults globally, and it is expected to increase frequently owing to three major factors as the aging population, rise in risk factors, and enhanced survival of post-MI [4, 5]. HF can be classified as either left ventricular systolic or diastolic dysfunctions, can also be called HF with reduced ejection fraction (HFrEF) or preserved EF (HFpEF) [6]. Patients with ejection fraction ≤40% are categorized as HF with reduced ejection fraction (HFrEF), and those with ejection fraction > or equal to 50% are termed as HFpEF. In both HFrEF and HFpEF, an increase in proinflammatory cytokines is predicted to worsen HF [7–9], which can be proposed that inflammation may add up to the development of disease in patients with HF.

Currently, macrophages have become a significant research area of interest under both normal and pathological conditions. Macrophage comprises the innate and adaptive immune system with its major role in defense of the immune system, inflammation, and tissue restoration. Monocyte which is known to play a vital role in the immune system protects the organs against harmful pathogens in a nonantigen-specific means either by direct removal of pathogens or by production of cytokines which includes tumor necrosis factor (TNF-alpha) and interleukin-1 (IL-1) and IL-2 [10]. Monocytes are considered to be the center source of inflammatory cytokines (TNF-alpha, IL-1 beta, IL-6, and IL-12), a main target of such cytokine with a minor quantity of chemokines being enough to recruit monocyte from the blood into various tissues and activate them to segregate into macrophages. It is known to play a major role in tissue inflammation as well as wound healing [11].

Modern methods and pharmacological remedies in present data have suggested a possibility to decrease infarct size, reduce death rate, and enhance contractile function in patients during and after MI [12, 13]. Resident cardiac macrophages are abundant in the mammalian heart; it increases in response to heart injury via circulating monocyte [14]. In MI, circulating blood monocyte migrates into the infarcted heart and differentiates into macrophages. Inadequate oxygen supply induces necrosis in the heart myocytes, which recruits inflammatory response. This inflammatory element is made up of neutrophils and macrophage penetration. Macrophages impact various wound healing processes, including the activation of the fibroblast which is vital for the formation of scar and also the activation of the endothelial cell which is important for angiogenesis [15]. For the past 30 years, inflammation has appeared as a therapeutic mark to reduce heart diseases. However, immunosuppression therapy has failed to progress the result after MI [16, 17] and HF [18]. These observations are coherent with the view that the functions of macrophages are vital in orchestrating repair of tissue and the resolution of inflammation [19]. In the early and late phases of heart disease, inflammation is considered as a major factor. Though the early broad-spectrum anti-inflammatory methods (thus, anti-tumor necrosis factor-alpha) did not demonstrate any particular therapeutic benefit in chronic HF [20, 21], newer evidence shows certain advantages of targeting specific inflammatory pathways for the treatment of HF [22]. During inflammatory response in acute or chronic cardiac injury, monocytes derived from the bone marrow and the spleen are attracted from the marginal circulation by chemotactic signals which are secreted from the disturbed endothelium and damaged tissue and move via the vessel wall into the tissue [23, 24]. Macrophages are however known to be the major contributors of inflammatory and fibrotic processes in HF [25–27]. A rising interest in the role of inflammation in the advancement of HF, mainly HFpEF, has been improved by the recognition that key comorbidity enhances an exaggerated systematic inflammatory response. However, the rise in inflammatory macrophage activity is connected to the growth of insulin resistance and diabetes which are common comorbidities in patients living with HF [28].

Macrophage controls several aspects of post-MI wound healing response and is considered as a therapeutic agent in HF; therefore, this review outlines a primary role of macrophages as an important regulator in cardiac injury and extracellular matrix in the late and early stage of heart disease. Finally, recent and future therapeutic approaches based on macrophage management for the treatment of MI and HF are discussed.

### 1.1. Development of Macrophages and Functions

Monocytes are white blood cells developed in the bone marrow from progenitor cells, and after development, they move from the bone marrow into the blood and circulate under homeostatic conditions for 1–3 days [29]. After the third day, they migrate into different organs where they form tissue macrophage (which plays major homeostatic functions in many organs) as well as giving rise to dendritic cells [30]. Macrophages play a multipurpose role in heart injury and wound healing by fibroblast activation and endothelial cells [31], and in most instances, they can self-regenerate by homeostatic proliferation [32, 33]. The self-maintenance of the monocyte was first studied in microglia which responded to various injuries, including central nervous system (CNS) damage and is capable of self-renewing without the involvement of blood-derived monocytes [34]. As evidence, in the CX_3_CR_1_ mice model, Yona et al. proved that tissue-resident macrophage which includes Kupffer cells and the lung, splenic, and peritoneal macrophages is recognized before birth and renews itself by proliferation during adulthood [35]. This finding was consistent with the discoveries by Schulz et al. [36], who concluded that the yolk-sac-derived tissue-resident macrophages are independent of Myb, which is a transcription factor required for hematopoietic stem cells (HSC) and monocyte development. These two studies in addition to many other research studies have proven that many tissue-resident macrophages are not regenerated from the monocyte steady state. The maintenance of the intestinal macrophage relies on the blood-derived monocyte [37, 38]. Macrophage is made up of two main phenotypes; the M1 (classical activated) and M2 (alternatively activated) macrophages. M2 macrophages are further divided into three subsets, namely, M2a, M2b, and M2c [39]. M1 macrophages in the initial stage of MI are responsible for the clearance of dead cells and matrix debris [40], and they produce numerous proinflammatory mediators which include cytokines and chemokines, thereby generating proinflammatory environment and gradually causing the enlargement of the infarcted zone in the heart [41, 42]. M2 macrophages, on the contrary, are developed after 5 days of MI to remove pathogens, prevent insulin resistance, and enhance cardiac remodeling and regeneration of cardiac tissues and are further dominated during the resolution of inflammation [43]. Cardiac macrophages are originated from yolk-sac-derived erythron-myeloid progenitors (EMPs) and are self-renewed in the steady state by local proliferation, yet in ischemic injury, these macrophages are replaced by blood monocytes [44]. Recent studies with genetic fate mapping demonstrated that tissue-resident macrophages in the brain, liver, lung, and skin are not generated from circulating monocytes but are replenished through local proliferation [35, 45]. CD14^++^CD16^−^, CD14^++^CD16^+^, and CD14^+^CD16^++^, which are named classical, intermediate, and nonclassical monocyte, respectively, have been identified in humans [46]. Classical monocytes are known to be vital scavenger cells which are made up of 80–95% circulating monocytes found to be highly phagocytic. Intermediate monocyte plays a major role in the production of ROS, antigen presentation and T-cell stimulation, inflammatory responses, and angiogenesis. These monocytes are made up of 2–8% circulating monocytes. The nonclassical monocytes are also involved in antigen presentation and T-cell stimulation, and they possess proinflammatory behavior which secret inflammatory cytokines following infections. These monocyte types move the endothelium in search of injury [47, 48].

### 1.2. Macrophages in the Normal Heart

**Figure 1:**
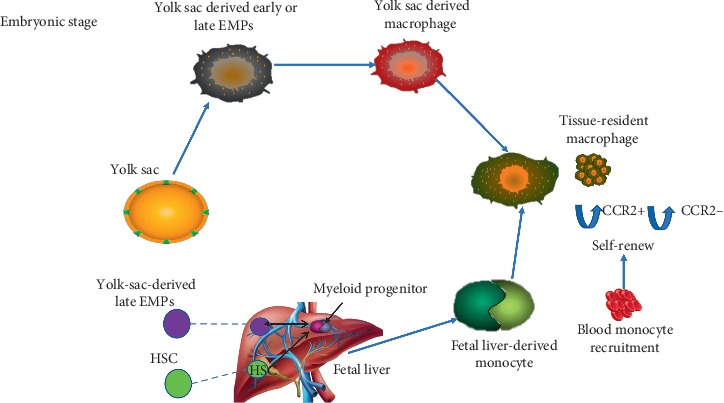
Macrophages in the normal heart.

It has been demonstrated in various literature studies that tissue-resident macrophages in the heart are established prenatally, continued throughout the life span, and regenerate themselves locally [49]. Scientists in many research studies described that one of the main components of the innate immune system is macrophages, and they play an essential role in cardiovascular disease [50, 51]. With age, the self-renewal property of the tissue-resident macrophages declines and allows only blood monocyte-derived macrophage to contribute to cardiac macrophage population and its advanced replacement by monocyte-derived macrophage even in the absence of inflammation [52]. Also, when it is disturbed from the steady state during sterile injury, a majority of cardiac macrophages are also derived from blood monocytes [14, 44], Figure 1.

The role of macrophages in a normal heart is more complicated because they generate into different functional phenotypes based on their microenvironment [53]. According to Pinto et al. histological analysis from the transgenic mouse model Cx_3_crl^GFP⁄+^ reveals abundant extravascular cardiac tissue macrophages (cTMs) in the healthy heart, however, in the heart of the adult mouse.

cTMs that are been established are described as macrophages which are located in the endothelial cell. cTMs are involved in the homeostatic function of tissue macrophages; cellular and molecular phases of cTMs describe a vital function for cells in cardiomyocyte homeostasis [54], which are located in the monocyte and endothelial cell. In this steady state, heart macrophages are anti-inflammatory, and the cells have a set of 22 genes which are associated with alternatively activated M2 macrophages which are translated at high levels and further express the surface marker Ly6C [54]. Added to this, the research by Frantz et al. [55] confirmed that there is a need to concentrate on heart macrophages because they perform various tasks in wound healing, tissue remodeling, and regeneration. There is a resemblance between cardiac macrophages in a healthy state and M2 macrophages. Cardiac macrophages express a plethora of the M2-designated markers [56] because M2 macrophages enhance the regenerating of tissue after injury and re-establish homeostasis [44]. A 2017 study by Hulsmans and colleagues revealed macrophage function in a healthy mouse heart using targeted macrophage reporter lines in combination with optical clearing techniques and confocal microscopy. They discovered that there are numerous macrophages in the atrioventricular (AV) node which interfere with cardiomyocytes via the connnexin-43-containing gap junction to accelerate myocyte repolarization and electrical conduction [57]. Cardiac macrophages are heterogenous in origin; they have been divided into four subsets with the use of different surface markers. These four populations which have been identified in the normal heart of a mouse express different levels of Ly6C, CD11c, CCR2, and major histocompatibility complex (MHCII) [58]. Of these subsets, Ly6C^−^ CCR2 forms the majority of which it originates from the yolk sac and contains MHCII^high^ and MHCII^low^ sets, and the third and fourth subsets are originated from hematopoiesis, and these are (Ly6C^+^ CCR2^−^) and (Ly6C^+^ CCR2^+^), respectively [14, 59]. The MHCII^low^ macrophage population has higher phagocytic capability and has the largest expansion in the cell number after cardiac stress is exerted. Using pulse labelling of macrophages indicated that embryo-derived macrophages to the MHCII^low^ subset were higher than MHCII^high^ macrophages. MHCII^high^ cardiac macrophages are more presenting function and outstanding antigen to T lymphocytes, however, decreasing with cardiac stress. On the contrary, CCR2^+^ macrophages express increased level of NLRP3-inflammasome via IL-1b-associated genes [14], and also, they are replenished by the bone marrow as well as CCR2^−^ are established in the developing mouse heart [14, 60], Figure 1. Studies by Heidt et al. analyzed a relatively small population of macrophages found in the heart of the adult mammalian of which it takes part in the immunosurveillance of myocardial tissue [44, 61]. The CCR2^−^ macrophage subset which is located in the myocardial wall and associated with coronary endothelial cells is necessary for the remodeling of the primitive coronary plexus via the secretion of proangiogenic signals [60]. However, the CCR2^+^ macrophages which are engulfed in collagen-rich scar tissues are enriched for genes known to enhance cardiac hypertrophy and inflammation [62].

Van Furth and Cohn in the late 1960s proved that tissue-resident macrophages are being developed from circulating bone marrow and spleen, but the fate mapping-studies recently disapproved the study. Genetic fate-mapping research demonstrated that tissue-resident macrophages are derived from the embryonic stage of yolk sac during the primitive hematopoietic phase [14] with less dependent on the blood-derived monocyte and are maintained by self-renewal. CCR2^+^ macrophages are being renewed by the recruitment of blood-derived local proliferation, while as CCR2^−^ macrophages are also renewed widely by local proliferation [14]. But, with age, the self-renewal of the resident macrophage declines hence allowing only blood monocyte-derived macrophages to contribute to cardiac population [52].

### 1.3. Macrophages in the Failing Heart

**Figure 2:**
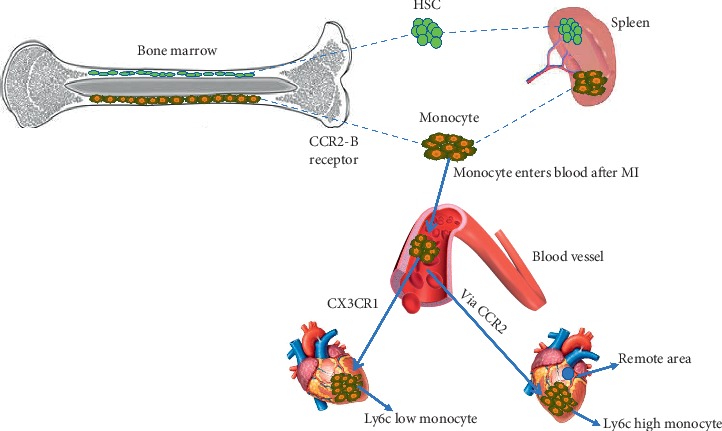
Macrophages in a failing heart.

During the first week of post-MI, numerous blood-derived monocytes enter the infarcted region which transform into macrophages [44]. Mortality rate increases in resident cardiac macrophages as they are recruited into the infarcted region. Within 24 hours after MI, macrophages are totally removed. In about 4 days, the number of macrophages which was removed within the initial stages regains its strength. At 8 weeks, macrophages increase to about 2.9 folds as a result of local macrophage regeneration and blood-derived monocyte recruitment [63], Figure 2. The infarcted heart tissue attracts the inflammatory Ly6C^high^ monocyte via CCR2^+^ within 30 minutes after induction of infarction through ligation of the left anterior descending (LAD) artery [64]; hence, these CCR2^+^ receptors help in promoting and regulating inflammation. The Ly6C^high^ monocyte cells are plentifully recruited from the bone marrow and spleen and accumulated in the infarct area, where this recruitment depends on MCP-1⁄CCR2 chemokine receptor interaction [64–66]. On the contrary, the Ly6C^low^ monocytes are recruited through CX_3_CR1 into the infarcted zone [67]. In the work of Swirisk and coresearchers, it was evident that the spleen's monocyte reservoir is released within 24 hours after MI [56], of which the splenectomy research reveals that the organ may add up to as much as half the monocyte population is recruited in the infarct zone within 4 days after MI, and the splenic monocyte reservoir refills by proliferation and differentiation of HSC and progenitors [41]. In 3–7 days after MI, tissue begins to regenerate by the recognition of phagocytic dying cardiomyocytes and neutrophils by macrophages which enhance anti-inflammatory and tissue-reparative cytokine production [68–70]. Inflammation resolves in days 7–14 after injury by the removal of debris and dead cells via cardiac lymphatic draining [24, 71]. Macrophages play a pivot role after myocardial injury, where there is an induction of CC chemokine in the infarcted myocardium which recruits abundant proinflammatory monocytes [66, 72], and these differentiate into macrophages [67] and exert phagocytotic actions. Macrophage vital roles are it triggers anti-inflammatory cascades and inhibits leukocyte recruitment [73]. During this stage, local proliferation in response to growth factor stimulation contributes to regenerating of the macrophage population in the healing infarcted area [44, 74]. Macrophage as a vital source of myeloid-derived growth factor (MYGDF), a growth factor that secretes proteins that promote the survival of cardiomyocytes, was identified in a recent study [75]. In addition to this, the release of inhibitory factors by the activated macrophage has been suggested to inhibit apoptosis of hypoxic cardiomyocytes *in vitro* [76, 77]. Experimental evidence suggested that, during the inflammatory phase of infarct healing, macrophage displays its role by clearing dead cells and matrix debris from the wound. Also, macrophage subsets may add up to the suppression and firmness of inflammation after the infarction, as well as specialized macrophage subset may promote scar formation and angiogenesis in the infarcted heart [78]. CCR2^+^ monocyte in the failing heart is been attracted by the cardiac fibroblast as well as CCL2 (chemokine C-C motif ligand 2) and granulocyte macrophages which are in the local production of chemokine and cytokines [79–81]. The attraction of CCR2^+^ monocyte occurrence leads to differentiation into macrophages [67]. The CCR2^+^ macrophage secrets proinflammatory cytokines in larger quantity of which it includes those associated with NLPR3 inflammasome which is important in interleukin (IL)-1*β* process to the heart during cardiac stress [14] in mice with lacking CCR2, and Ang-II and IL-1*β* production is blocked [82, 83]. Furthermore, neutralizing antibody knockdown CCR2 in the bone marrow cells has effects on cardiac hypertrophy during Ang-II infusion and pressure overload [84]. DAMPs (damage-associated molecular patterns) which include adenosine triphosphate (ATP) and self-DNA recognition released by dying cardiomyocyte irritate the responses of proinflammation from macrophages [85, 86] causing tissue damage. Other scientists revealed that the rapid accumulation of inflammatory cells is as a result of ischemic injury leading to acute death of myocytes [87].

In the adult stage, monocyte is derived from the bone marrow and spleen which enters the blood vessels through the CCR2-*β* receptor after MI; this monocyte is recruited into the infarcted zone via CCR2. Thus, Ly6C^high^ monocyte enters the infarcted zone, while the Ly6C^low^ monocyte is recruited into the infarcted zone via CX3CR1. Death occurs in resident cardiac macrophages as they enter the infarcted area within 24 hours of MI, in about 4 days after a number of macrophages regain their strength and begin to increase in number. At week 8, the macrophage number increases to a 2.9-fold as a result of local and renewal of blood monocyte recruitment [63]. In the infarcted heart, there is a noninfarcted remote area which shows changes in inflammation and macrophage number after MI. A study by T.A Ramirez revealed that within 4 weeks of post-MI, the remote zone contains more inflammation than the infarcted area [88].

### 1.4. Macrophage Production and Functions after Myocardial Infarction (MI)

Hematopoietic stem cell (HSC) from the bone marrow which enters the spleen activates extramedullary hematopoietic and the production of monocytes [89]. This splenic monocyte reservoir is released within 24 hours after MI [56] and replenished within 4 days after MI by proliferation and differentiation of HSC and progenitors [41]. Though proliferation of HSC in the spleen is stem cell-factor-dependent, the reduction of HSC proliferation and monocyte production is as a result of stem cell-factor neutralization [89]. Added to this, the splenic monocyte production has been noted to be dependent on interleukin-1*β* [41], interleukin-3, and granulocyte-macrophage colony-stimulating factor (GM-CSF) [23]. Furthermore, following acute MI, the Ly-6c^high^ and Ly-6c^low^ monocyte subsets are found to be significant in cardiac healing process [89]. Thus, whereas the Ly-6c^high^ macrophage produces early inflammatory macrophages and removes all dead tissues and necrotic debris by phagocytosis and proteolytic enzyme secretion, the Ly-6c^low^ macrophages in the second phase enable wound healing and cardiac regeneration by promoting the accumulation of myofibroblast, collagen deposition, and angiogenesis [42]. Monocytes that enter the infarcted heart might inter-relate with the extracellular matrix in the damaged heart which is consequential to the release of fibronectin [42]. The infarct heart fibronectin stabilizes and decreases infarct rupture. Once fibronectin enters the infarct myocardium and stabilizes it, monocyte separates into macrophages in the presence of M-CSF [90].

Macrophages have also been reported to play significant roles in organ renewal. Although MI in adult mammals' heart leads to scarring and reduces the roles of the left ventricular heart, regeneration of the infarcted heart after MI without damaging only happens in the myocardium of the neonatal mouse [91]. But, this repair process can only be delayed when cardiac macrophages are depleted. A recent study by Lavine et al. [92] demonstrated that angiogenesis and healing of the infarcted heart after damage are promoted by cardiac macrophages derived from the early embryonic cells [93]. Although inflammation is required for the clearance of the dead matrix and regeneration of new tissue after ischemic injury, the healing process of the ischemic heart can be obstructed when inflammation is exaggerated [94]. As a proof, in Mcp-1-deficient mice, there was decreased recruitment of monocyte in the infarcted heart [66]. Though the infarct sizes for the Mcp-1-deficient and wild-type mice were almost the same, the Mcp-1-deficient mice had their ventricular heart function enhanced, hence showing the relevance of monocyte in the repair of the infarcted heart following MI [90].

### 1.5. Extracellular Matrix (ECM) Remodeling in Macrophages

In response to myocardial infarction (MI), cardiac macrophages regulate inflammation and scar formation. Myocardial infarction (MI) invokes a cardiac wound healing response that involves early initiation of inflammation followed by robust scar formation in the infarct area. The macrophage is a key regulator of cardiac remodeling, providing both strong proinflammatory signals early and reparative cues later [95]. Although monocyte- and macrophage-derived molecules are known to promote extracellular matrix (ECM) disruption and destabilization, it is less appreciated that they also synthesize molecules contributing to ECM formation, stabilization, and function [96].

ECM contains various proteins which aid the system cells. Its structure is complicated and dynamic. It does not simply play a role as a mechanical scaffold to produce cellular and acellular networks within the heart but may also transduce key signals that are vital for the survival and function of the cell. ECM in the heart comprises two subsets, i.e., the interstitial matrix and the basement membrane. The interstitial matrix is made up of primarily type I and type III collagen, while the basement membrane comprises collagen IV, V, VII, and X and laminins [97]. ECM degradation is important for the repair of damaged tissues, and its activation in the heart occurs in the first 10 min of myocardial infarction [98]. Matrix metalloproteinases (MMPs) which are generated by macrophages and fibroblasts are secreted as part of a programmed inflammatory response in order to analyze the matrix structure.in the development of MI; various cardiomyocyte necroses emphasize on matrix degradation. ECM dynamics ranging from the native to the plasma-derived and then cell-derived remodeled matrix is an ordered process to allow for efficient transition from the inflammatory response to wound repair. Any irregularities in this process can result in inflammation and fibrosis. A newly ECM generated is different from the original native ECM, with turnover of cross-linked collagen being rapid than that of normal collagen [99]; this results in the stiffness of collagen fibers and eventually stiff scar tissue in post-MI [100]. Scar tissue formation in post-MI is necessary for the sustaining of structural integrity while the heart is under reconstruction, and wide scarring or remodeling limits the functional capacity of the heart by impeding ventricular contraction and relaxation [101]. Furthermore, the damaging effect of ECM remodeling goes beyond the infarct site as formation of scar peripheral to the site of infarction is also observed. Both proinflammatory and anti-inflammatory macrophages play a distinct pathological role in ECM remodeling, yet both subsets also have vital roles in natural healing and repair. Therefore, it is difficult to pinpoint precisely which subset is a therapeutic target without further delineation of their functions in the infarcted heart [102–104]. Metalloproteinases are not limited to ECM breakdown; such enzymes have a role in regulation of the inflammatory response through proteolytic cleavage of cytokines, chemokines, and growth factors [105].

### 1.6. Inflammation in Postischemic HF

Pharmacological studies established that the flow of blood in the coronary artery can save the ischemic heart from death and preserve the functions of the heart. Cardiac reperfusion in the primary stages of MI is believed to induce injury through the activation of inflammatory pathways [12]. The generation of reactive oxygen species (ROS) and the release of cytokines into the ischemic heart promote the recruitment of neutrophils via a loop [106]. Neutrophil penetration into the injured heart resolves through cell apoptosis in 3 to 7 days after MI [73]. The resolution of neutrophil inflammation is a dangerous step for ischemic repair process, and numerous inhibitory signals have progressed for the negative guideline of inflammatory cascade following heart injury [107–109]. However, neutrophil-mediated inflammation aids itself in infarct healing. The clearance of dead cells from the infarcted heart is advantageous for MI healing, and this is mediated by M2c macrophages thus even 4 to 7 days after MI. M1 macrophages that are neutralized by the phenotype in 1 to 3 days after MI are characterized by the release of high levels of IL-10 and transforming growth factor-*β* (TGF-*β*) [64, 110]. In the absence of neutrophil secretome, especially neutrophil gelatin-associated lipocalin (NGAL), there is ineffective dead debris clearance as a result of impaired macrophage phenotype shift [111]. Macrophages enter the nonischemic heart after MI but more slowly as compared to the rate at which they enter the ischemic heart, thus reaching to its expected end at day 10 after ischemic injury [63, 112]. A low-grade inflammation state may add up to the disturbance of the extracellular matrix via the proteolytic activity of matrix metalloproteinases and cathepsin, whereas the importance of inflammatory macrophage activation in cardiomyocyte apoptosis is an interesting theory but needs wider studies [113]. Once MI occurs, safeguarding inflammatory response made by macrophages may increase a range of cytokines which are important for short-term adaption to stress; the cytokine theory proposes that HF improves at least in part as a result of deleterious effect applied by endogenous cytokine cascade on the myocardium and the peripheral circulation [114].

### 1.7. Inflammation and Macrophage Activators

A recent research conducted by Gomez et al. and Hoeffel G. et al. demonstrated that yolk-sac EMPs which expand in the fetal liver are common sources of macrophages in adult tissues [115, 116]. According to these findings, cardiac macrophages have been originated to a level that different phenotypes and roles of the yolk sac are formed by the local environment of the resident tissue [117]. There is relatively an unknown extent on how long the yolk-sac-derived macrophage lives in the adult tissue [118], and also, the number of yolk-sac-derived macrophages seems not to be permanent in the heart of a mouse as it deteriorates with age due to the fact that, as the mouse is aging, rate of proliferation of cardiac macrophages reduces and becomes inadequate for the resident macrophage to be sustained [52]. During the progression of cardiac reperfusion, macrophages are actively involved in the inflammatory response [64], and since the main role of the macrophage is to clear debris after MI, the circulating monocyte is recruited speedily to the infarcted myocardium to involve in debris clearance, wound healing, angiogenesis, and the regeneration of the tissue. Left ventricular heart remodeling and HFrEF dysfunction are induced by damages within the myocardium. Heart failure starts with intramyocardial inflammation arising after cardiomyocyte cell death from ischemia, reperfusion injury, or genetic mutation [119]. This inflammation enhances the replacement of myocardium with noncontractile fibrotic scar which all leads to HFrEF [120]. Inflammation has been emerged as a therapeutic target to mitigate cardiovascular diseases for some couple of decades ago. Moreover, various strategies using broad immunosuppression have failed to improve the end result of MI [16, 17] and as well during HF [18]. Hence, these observations are accurate with the idea that immune function is fundamental in orchestrating the repair of tissue and inflammation resolution. Heart inflammation can be characterized by both local cell death, loss of CCR2^−^ macrophages, and their replacement by recruited CCR2^+^ macrophages [19]. In the infarct region, the initial inflammatory phase from day 0–2 is been characterized by 50% decrease of resident macrophages [121].

### 1.8. Macrophages and Inflammatory Biomarkers

In the pathogenesis and advancement of different kinds of HF, inflammation is an important factor, and biomarkers of inflammation have now become a significant research area to deal with [122]. The presence of inflammatory cells such as macrophages derived from monocyte and T-lymphocyte at the site of rupture is proceeded by dysfunction of activated endothelial cells which generate adhesion molecules that interact with inflammatory cells [123–125]. According to Guillermo et al. macrophages secrete cytokines such as TNF, IL-6, IL-8, and IL-12. Despite the main sources of these cytokines being monocyte and macrophage, they are actually produced by activated lymphocytes, endothelial cells, and fibroblasts [126]. The fate of macrophage is been biased by cytokines into a spectrum of inflammation promoting M1 or M2 macrophages. These cytokines are released from the myocardium, lung, liver, leukocyte, platelets, endothelial cells, and other cell types. Also, cardiomyocytes are an important source of proinflammatory mediators that help in the elevation of HF [127, 128]. Once cytokine release has been initiated and inflammation is triggered at the beginning of atherosclerotic lesion development, numerous factors are found in the atherosclerotic plaque which participate in maintaining and amplifying the production of cytokines which include adipokines, angiotensin II, heat shock protein (HSP) immune complexes, ROS [129], and proinflammatory cytokines. Ever since the initial observation by Levine and other researchers [130, 131], numerous studies have demonstrated a correlation between elevated circulatory levels of proinflammatory cytokines and adverse clinical outcomes in HF [132–134]. Both TNF and IL-1 may induce dysfunction of the cardiac muscle by a variety of mechanisms [135, 136]. The activity of inflammatory cytokine is also enhanced by anti-inflammatory cytokines such as transforming growth factor-*β* (TGF-*β*) and interleukin-10, which can downregulate the formation of several kinds of inflammatory cytokines from macrophages and other cells. There are some therapeutic approaches to treat inflammatory disease, and these include monoclonal antibodies that either neutralize inflammatory cytokines or their receptors [137]. The zeal in studying the role of inflammation in HF has been dampened because of the disappointing results of targeted anticytokines [135], and due to this failure, researchers have continued studies and had an in-depth understanding of the role of inflammation as well as the identification of the current biomarkers such as sST2 (somatostatin receptor subtype 2), galectin-3, and pentraxin-3, which have given a new insight with respect to the diagnosis and prognosis of HF patients. Inflammatory response in HF is closely intertwined with the activation of the immune system, which is demonstrated by elevated circulatory levels of inflammatory cytokines such as IL and TNF superfamily (TNFSF), and members of IL-1 and IL-6 are all proinflammatory cytokines that are found in the HF [138]. Inflammatory mediators that have garnered enough attention, including galectin-3 and pentraxin-3, can be referred to as macrophage biomarkers. Macrophages in response to tissue injury release galectin-3 which plays a vital role in fibroblast activation leading to tissue fibrosis formation as well as pentraxin-3 which is an inflammatory marker found in the HF patient, but unfortunately, its role is not known. The severity of HF in patients is increased due to the circulating levels of TNF and TNFSF, IL-6, IL-18, and IL-33 [139].

### 1.9. C-Reactive Protein (CRP)

CRP is a protein that is found in serum in various inflammatory conditions [140]. It is produced by the liver in retorts to stimulation with proinflammatory cytokines and a useful biomarker that can be used to predict the result and progression of HF in patients, as well as the cardiac rupture of patients suffering from MI are being predicted using the increasing levels of CRP [141, 142]. Research has further noticed that the increasing levels of CRP are a sign of inflammation in patients suffering from HF [143]. High sensitivity levels of CRP (hsCRP) are linked to the long-term result of HF independently of natriuretic peptides [144, 145]. HF patients with a final stage of the disease have 8-fold higher levels of circulating CRP than the healthy patients with a reference value of 0–5 mg/L in serum [146]. To improve the inflammatory profile of HF patients that have high levels of CRP, the profile levels should decrease to the normal range of 0–5 mg/L in an interval of 60 days after implantation surgery [147].

### 1.10. Tumor Necrosis Factor-Alpha (TNF-Alpha) System

Dilated cardiomyopathy which is a disease that usually starts in the left ventricles of the heart is induced by TNF-alpha through the matrix of metalloproteinase activation [148], and as well, myocyte apoptosis and necrosis are been caused by proinflammatory cytokines [149]. In some years to come, the development of HF in elderly will be predicted by IL-6 and TNF-alpha [148]. Despite the discontinuation of the anti-TNF-alpha treatment in patients with HF due to the supposition that it does not confer any positive effect in HF patients [135, 140], a rise in plasma levels of TNF-alpha is associated with an increased death rate [150]. The cardiac expression TNF-alpha and IL-6 is induced by pressure overload [151, 152]. Patients with reduced serum levels of IL-6, IL-8, TNF-alpha, and TGF-beta indicate that the patients respond to cardiac resynchronization therapy [153]. Hence, more research is warranted to better understand the reason for the failure of the anti-TNF therapy and perfectly tailor therapy for the treatment of inflammation-associated HF.

### 1.11. Fas/APO-1

Fas is activated by the apoptosis signal from the Fas ligand (FasL) and plays a vital role in HF development, where high serum levels of Fas found in patients with HF indicate the severity of the disease [154]. The decrease in postinfarction ventricular models in animals is been inhibited by soluble Fas in the animals as it enhances survival [155]. In patients with ischemic HF, functions of the left ventricle are been enhanced due to the reduction of Fas and CRP by an immunomodulating agent such as pentoxifylline [156] or intravenous immunomodulating [21]. Therefore, therapeutically targeting Fas in HF treatment might portend enhance prognosis.

### 1.12. M1-Macrophage Polarization and Its Role in Inflammation

Macrophage expansion population occurs via local proliferation and monocyte recruitment during cardiac stress [14, 67]. They are powerful effector cells of the innate immune system and are vital in the removal of debris and tissue repair. Human studies of the M1/M2-like macrophages show both routes of induction and its biological process which is regulated and do not fall within such a common schema; upon environmental changes, the original polarization can be reversed [157, 158]. The M1/M2-like macrophages have become an interesting research area of study because most studies were interested in the identification of markers which can differentiate between the M1/M2 macrophages, of which they can play an important role in determining the activation status of human macrophages and inflammation [159, 160]. Upon the dynamics in the microenvironmental conditions, human monocyte is polarized to the M1-like phenotype and then switched to M2-like macrophages and vice versa [161, 162]. Over a period of time, M2 and M1 macrophages lose their polarized phenotype in a medium free of cytokines; by day 12, polarized macrophages are reversed to an unattached macrophage state in a medium lacking cytokine. After 6 days resting, in a cytokine-deficient medium, there is a switch in macrophage polarization when macrophages are given another polarized stimulus. There is a comparable change in the phenotype of M1 and M2 macrophage cells. With IL-13 treatment, M1-IFN-gamma reverts to CD11b^+^ CD209^+^ M2 macrophage, and also, with IFN-gamma treatment, there is a change from M2 to M1 macrophage. Therefore, switched M1-like macrophage loses its endocytic activity, but its phenotypic activity is not lost, as well as M2 cells attains their phagocytic activity [160]. The first line of defense against intracellular pathogens comprises the M1 macrophages which enhance the Th1 polarization of CD4 cells as these macrophages occur in an inflammatory environment which is dominated by toll-like receptors (TLR) which evoke treatments *in vitro*. The type of TLR ligands is the bacteria lipopolysaccharides and interferon (IFN) signaling, and most protocols use GM-CSF or type II IFN and TLR agonists for polarization in the M1 macrophage [163–165]. CD64 and CD80 are the best two markers that characterize M1 macrophages despite the level of expression of these markers being dependent on the nature of M1 stimulus [160]. M1 macrophages have the ability to guide acute inflammatory response and are able to secrete high levels of proinflammatory cytokines and several chemokines. However, to increase their pathogen-destroying ability, they produce a high amount of ROS and nitrogen radicals. The CX3CL1 chemokines induce Th1 response activation, thereby facilitating a complement-mediated phagocytosis and type 1 inflammation [159, 166–168].

### 1.13. Macrophages in the Early and Late Phases of Inflammation after MI

In the early stages of inflammation, the left ventricular heart regains its stability which is produced by a new matrix in the production phase, and during this stage, there are less abundant Ly6C surface markers [64]. Macrophages that are active during the early stages become less inflammatory, expressing genes that are connected with M2 macrophages [169]. These M2 macrophages aid in revitalizing the tissue. On the safer side, it is assumed that the Ly6C^high^ monocyte subset gives rise to the inflammatory M1 macrophage in the earlier days after MI; likewise, in the kidney, Ly6C^high^ monocyte that is recruited into it changes into M1 macrophages in the early stages of inflammation, but these recruited monocytes differentiate into M2 macrophages when inflammation is declining at the later stage [170]. A recent study by Van der laan [40] verified using the dead bodies of patients suffering from MI to perform an autopsy, which was reported that CD14+CD16+monocyte was found in the infarct border zones of patients who died later on. The early phase after MI is recruited by Ly6C^high^ macrophages as these Ly6C^high^ inflammatory macrophages generate a cardioprotective function which facilitates phagocytic cell debris in acute inflammation [171]. A research work proved that, in the early stage of infarction, there is a wide inflammatory response which is been accompanied by the fibrotic scar deposition at the late phase of injury [172].

### 1.14. Depletion of Macrophages in the Infarcted Heart, Beneficial or Nonbeneficial?

Macrophages have been described as beneficial components in the heart as they help in the clearance of debris from the heart, but when they are being depleted by clodronate liposome injection during the earlier stages after MI, what happens to the heart? Is it beneficial or harmful?

In Frantz review, it was explained that the damage of the macrophage can lead to the attachment of left ventricular mural thrombi to the infarcted area which may be beneficial to patients with left ventricular thrombus as it will show a reduction of the CD14^+^ and CD16− monocyte subset in the blood [55]. However, this can also lead to the increase of necrotic cell debris presence of neutrophil [14, 78] and damaged extracellular matrix from the infarcted zone, thereby attracting other immune cells through chemokine and proinflammatory secretions. On the contrary, early stages of macrophage/monocyte depletion worsen wound healing effects [173], as well as left ventricular (LV) remodeling irritation after MI. Macrophages vanish in about 2 to 3 weeks after MI as the granulate tissue develops into a solid scar; during this phase, the recuperating heart changes ventricular functions [174, 175]. Though the main role of the macrophage after MI is not understood vividly [176], after MI, macrophages are needed for the wound-healing response, but when these macrophages are inhibited by injection of liposome-encapsulated clodronate, it results in a decrease in wound healing effect with complications following the break of left ventricular or a formation of left ventricular thrombi [177]. The clodronate binds intracellular adenosine triphosphate (ATP) and prevent ATP to perform its roles resulting in cellular apoptosis [95]. Numerous pharmacological studies have reported the effect of injecting liposome clodronate into hypertensive rats (Ren2rat) in other to deplete macrophages in the infarcted zone and induce the CD4^+^ T-cell-dominant inflammatory cell [178]. In view of this, in the early stages of depletion, the cardiac contractility is reduced, resulting in the protection of myocardium by the cardiac macrophages against hypertensive-induced stress responses [178].

The study by Pipp and colleagues [179] suggested that the depletion of macrophages can lead to the decrease of growth factors and also in neovascularization, which reveals that, in myocardial wound repair, macrophages are the essential regulators of vessel formation. Macrophages demonstrate a higher TGF-beta level in the infarct area as this TGF-beta is best known to induce the fibroblast to the myofibroblast after myocardial injury; therefore, the depletion of these macrophages leads to a low availability of myofibroblast in the cryolesions, signifying that macrophages are important cells for the formation of myofibroblast and the recruitment after myocardial injury [180]. HSC in the bone marrow is been reserved by CD169^+^ macrophages; hence, the depletion of these macrophages damages the regeneration of the red blood cell [181].

### 1.15. Macrophages as a Therapeutic Target in MI

Despite the great successes by pharmacologist and other researchers over the past three decades, there is still no best method that affects myocardial healing, but other interventional therapies have helped decrease the mortality rate in patients with acute MI [182, 183]. Currently, the most vital and complex task in modern cardiology is the hunt for the therapeutic target that is capable of preventing, limiting cardiac remodeling, and interfering the growth of left ventricular dilation [184]. In several ways, macrophages control cardiac remodeling and healing after MI via protease secretions, growth factors, and proliferation [184]. Macrophages are attractive targets for therapeutic activities because they are helpful in several pathological processes [185]. Due to the high plasticity effects of macrophages, they play a vital role in inflammation resolution and also have the ability to dampen inflammation and enhance extracellular matrix regeneration and cell proliferation in the late stage of MI [67]. CD206 ^+^F4/80^+^CD11b^+^ are alternative M2 macrophages recognized in the heart of murine, indicating the healing of the infarcted heart as a result of their fibroblast activation function [186]. Growth differentiation factor-15 (GDF-15) produced in the infarcted zone of the myocardium plays a vital role in regulating the recruitment of inflammatory cells. This is done by limiting the infarcted zone by blocking monocyte attraction to the inflammatory-mediated infarcted area through decreasing the rupture of the left ventricle [187, 188]. The blockage of the inflammatory monocyte can be obtained by chemokine exposure resulting in a reduction of circulating inflammatory monocyte thus elimination of the CCR2 receptor; hence, in order to gain a positive effect on cardiac remodeling, the decrease of the CCR2+ monocyte can be attained as a result of inflammatory response after MI [189, 190]. During cardiac repair, B-lymphocyte cells which contribute to the recruitment of the Ly6C^high^ monocyte in the infarcted zone by secreting CCL7 interact with the monocyte. Therefore, the depletion of these B-lymphocyte cells using the CD20-specific monoclonal antibody [191] leads to the reduction of monocyte and Ly6C^high^ monocyte in the tissue resulting in the reduction of the infarcted area [192, 193]. The inhibition of nuclear-factor-kappa-B (NFkB) improves the functions of the heart and the survival of cardiomyocytes through cytoprotective program activation against MI [194]. Various research studies have proved that the activation of signal transducer and activator of transcription 1 (STAT1) contributes to cell death, while STAT3 is associated with cardiac protection after MI [195]. Another therapeutic target for MI treatment is peroxisome proliferator activator receptor (PPARγ) which protects the heart via inflammation suppressing and enhancing the metabolism of glucose and lipid [196]. Cardiac dysfunction and fibrosis in MI of rats would be better with 5-azacytidine (5-AZ) because 5-AZ helps M2 macrophage polarization by way of inhibiting iNOS [197]. Furthermore, research has revealed the interferon regulatory factor-1 (IRF-1)-dependent mechanism by which the phenotype in macrophages towards cardio protection is been induced by 5-AZ [198]. Also, the muting of IFR5 which is a regulator of macrophage polarization is shown to be involved in cardiac remodeling, decreasing inflammatory macrophages, and enhancing infarct healing [199].

Along the line, Di Filippo established that the only way to decrease the injured size and recover left ventricular ejection fraction after 25–30 minutes of ischemia and 2 hours of reperfusion is by preadministration with telmisartan in Zucker diabetic fatty rats [200]. Telmisartan upgrades M2-specific cytokine and chemokines. Another therapeutic target against MI which was uncovered by Tian et al. is the role of BAY 60–6583 which reduces myocardial infarct size in C57BL/6 mice after 40 min ischemia and 1 hour of reperfusion and also decreasing the infiltration of M1 macrophage neutrophils as well as increasing the accumulation of M2 macrophages in the perfused myocardium via P13K/AKt pathways [201].

## 2. Conclusion

This review summarizes on the roles and therapeutic measures presented by the study of cardiac macrophages. In the past decades, scientists have researched on inflammatory and tissue macrophage in various organ systems, but very little is known about cardiac macrophages, possibly because rapid cardiac motion has made it difficult to study these macrophage cells *in vivo*. In modern science, advanced imaging tools have now paved way for researchers to investigate into macrophage fate, numbers, and function at different scales in the healthy mouse heart to the infarcted zone. Currently, macrophage-specific gene knockouts and macrophage ablation approaches are used to investigate more into the role of macrophages in heart diseases. Macrophages are vital cells in the innate immune system and are implicated in various forms of cardiac diseases. HF in its early phases is involved in host defense by removal of inflammatory ligands, phagocytosis, and necrotic debris. Monocytes are involved in both tissue injury and repair; a disproportion of this balance in HF is likely to be significant in disease development. Finally, the activation of macrophages plays a pivot role in inflammatory pathophysiology of HF and occurs through extensive stimuli, many of which are ill explained. The release of inflammatory cytokines, relocation to the heart, bonding to the endothelial wall, and penetration into the heart are complex processes involving an interaction between numerous components of the immune system. This level of complexity would better explain reasons why therapeutic modulation of inflammation and macrophages has yet not been globally successful in the treatment of HF among many clinical trials.

## Abbreviations

5-AZ: 5-Azacytidine
Ang-II: Angiotensin II
APO: Apoptosis antigen
ATP: Adenosine triphosphate
AV: Atrioventricular
CCL-2: Chemokine C-C motif ligand 2
CCR2: Chemokine C-C motif receptor 2
CHF: Chronic heart failure
CNS: Central nervous system
CRP: C-reactive protein
DAMPs: Damage-associated molecular patterns
DNA: Deoxyribonucleic acid
GDF: Growth differentiation factor
GM-CSF: Granulocyte-macrophage colony-stimulating factor
HF: Heart failure
HFpEF: Heart failure preserved ejection fraction
HFrEF: Heart failure with reduced ejection fraction
HSC: Hematopoietic stem cells
hsCRP: High sensitivity levels of CRP
HSPs: Heat shock proteins
IFN: Interferons
IL: Interleukin
iNOS: Inducible nitric oxide synthase
IRF: Interferon regulatory factor
LAD: Left anterior descending artery
LV: Left ventricular
M1: Classical activated macrophages
M2: Alternatively activated macrophages
MCP-1: Monocyte chemoattractant protein-1
M-CSF: Macrophage colony-stimulating factor
MHC II: Major histocompatibility complex
MI: Myocardial infarction
MYGDF: Myeloid-derived growth factor
NFkB: Nuclear-factor-kappa-B
NGAL: Neutrophil gelatin-associated lipocalin
PPAR*γ*: Peroxisome proliferator activator receptor
ROS: Reactive oxygen species
SST2: Somatostatin receptor subtype 2
STAT1: Signal transducer and activator of transcription 1
TGF: Transforming growth factor
Th-1: T-helper type 1
TLR: Toll-like receptor
TNF: Tumor necrosis factor
TNFSF: Tumor necrosis factor superfamily.
